# Pregabalin, the lidocaine plaster and duloxetine in patients with refractory neuropathic pain: a systematic review

**DOI:** 10.1186/1471-2377-10-116

**Published:** 2010-11-19

**Authors:** Melanie Plested, Sangeeta Budhia, Zahava Gabriel

**Affiliations:** 1 Heron Evidence Development Ltd, Butterfield Technology Park, Luton, UK; 2 Pfizer Ltd, Walton Oaks, Dorking Road, Walton-On-The-Hill, Surrey, UK

## Abstract

**Background:**

Patients frequently fail to receive adequate pain relief from, or are intolerant of, first-line therapies prescribed for neuropathic pain (NeP). This refractory chronic pain causes psychological distress and impacts patient quality of life. Published literature for treatment in refractory patients is sparse and often published as conference abstracts only. The aim of this study was to identify published data for three pharmacological treatments: pregabalin, lidocaine plaster, and duloxetine, which are typically used at 2^nd ^line or later in UK patients with neuropathic pain.

**Methods:**

A systematic review of the literature databases MEDLINE, EMBASE and CCTR was carried out and supplemented with extensive conference and grey literature searching. Studies of any design (except single patient case studies) that enrolled adult patients with refractory NeP were included in the review and qualitatively assessed.

**Results:**

Seventeen studies were included in the review: nine of pregabalin, seven of the lidocaine plaster, and one of duloxetine. No head-to-head studies of these treatments were identified. Only six studies included treatments within UK licensed indications and dose ranges. Reported efficacy outcomes were not consistent between studies. Pain scores were most commonly assessed in studies including pregabalin; trials of pregabalin and the lidocaine plaster reported the proportion of responders. Significant improvements in the total, sensory and affective scores of the Short-form McGill Pain Questionnaire, and in function interference, sleep interference and pain associated distress, were associated with pregabalin treatment; limited or no quality of life data were available for the other two interventions. Limitations to the review are the small number of included studies, which are generally small, of poor quality and heterogeneous in patient population and study design.

**Conclusions:**

Little evidence is available relevant to the treatment of refractory neuropathic pain despite the clinical need. There is a notable lack of high-quality comparative studies. It is evident that there is a need for future, high quality trials, particularly "gold-standard" RCTs in this refractory patient population.

## Background

Neuropathic pain (NeP) is defined as "pain initiated or caused by a primary lesion or dysfunction in the nervous system" [1]. Patients experience pain described most frequently as burning, tingling or electric shock [2]. It is generally persistent and/or chronic in nature and can be further categorised as either peripheral or central depending on the origin of the lesion or dysfunction [1,3,4]. The type of pain experienced varies considerably between NeP sub-types and within sub-types. Pain may be present with or without a stimulus continually (though intensity may vary) or intermittently [5].

NeP is a widespread condition, with an annual incidence of almost 1% of the general population [6]. Triggers for NeP include cancer, diabetes and HIV infection, as well as surgery, radiation and inflammation [5]. Common NeP syndromes include diabetic peripheral neuropathy (DPN), trigeminal neuralgia (TN) and complex regional pain syndrome (CRPS). NeP is more common in women and incidence increases with age [6]. The prevalence of NeP is expected to rise due to population aging and the increased longevity of patients with cancer, HIV-infection, diabetes and other diseases [5].

Resistance, insensitivity or intolerability to first-line treatments is common [7]. To the authors' knowledge, there are no published definitions of refractory NeP, but there is a proposed definition of pharmacoresistant NeP: "A neuropathic pain condition is resistant to pharmacotherapy when mono-or a rational combination treatment using drugs proven efficacious in RCTs fails in inducing useful pain relief from the patient's/physician's point of view after an appropriate duration of treatment with adequate dosage, or if intolerable side effects occur" [8]. Owing to the lack of a consensus definition of refractory NeP, this review took a pragmatic approach to define refractory NeP more broadly as patients who had failed to receive adequate pain relief from or were intolerant to previous therapy irrespective of the duration, dose and type of previous therapy.

Persistent pain syndromes cause suffering and psychological distress in association with reductions in quality of life [9]. Despite this clinical need, there are few published studies on refractory pain, and these are frequently reported as conference abstracts rather than in full peer-reviewed publications, which may be difficult for clinicians to locate. Recent reviews of treatment efficacy in specific NeP conditions, have offered little focus on the refractory patient setting [10-12]. The reviewers predicted that the class of evidence would be low, but aimed to identify and summarise this literature in one review to ensure accessibility of this clinically valuable data. The aims of this review were:

a) to identify the evidence base in refractory neuropathic pain for three pharmacological treatments (pregabalin, lidocaine plaster and duloxetine) which are typically used at 2^nd ^line or later in UK patients with neuropathic pain, and

b) to determine the efficacy, safety and tolerability of these drugs in this refractory patient population.

## Methods

### Literature searches

A comprehensive search of the major literature databases Medline (In-process and other non-indexed citations), Embase and the Cochrane Clinical Trial Registry was undertaken. The databases were searched from 1st January 1998 to 12th December 2008. The comprehensive search strategy, which was aimed to retrieve all studies in neuropathic pain and was not restricted by refractory terms, can be found as an additional material to this publication (additional file 1). In addition, abstracts from eleven conferences (World Congress of Pain, NeP Specialist Interest Group (NeuPSIG), British Pain Society, European Congress on Neuropsychopharmacology, American Academy of Neurology, European Federation of Neurological Societies, European Neurological Society, American Society of Anaesthesiologists, European Society of Anaesthesiologists, World Congress of Anaesthesiologists, Congress of the International Anaesthesia Society) were hand-searched between 2004-2008. The OpenSIGLE (System for Information on Grey Literature) and Google Scholar databases were also searched using multiple keyword searches to identify additional studies published in the disease area; approximately 3500 and 5500 references were reviewed in the Open SIGLE and Google scholar databases respectively.

### Inclusion/Exclusion criteria

Studies that enrolled adult patients with refractory NeP (central or peripheral) due to any cause, or lower back pain with a neuropathic component were included (Table 1). It was predicted that the evidence base for the review would be limited. Therefore, studies of any design, quality or sample size (except case studies for one patient) were included. Owing to the lack of a consensus definition of refractory NeP, this review took a pragmatic approach to define refractory NeP broadly as patients who had failed to receive adequate pain relief from or were intolerant to previous therapy irrespective of the duration, dose and type of previous therapy. Due to the limited number of studies available, all studies assessing 'refractory' patients using alternative definitions or where undefined were also included in this review. Studies enrolling a mixed population of refractory and non-refractory patients were included. All studies of refractory patients were included irrespective of number or type of previous treatments or duration of previous treatment. Included interventions were pregabalin, the lidocaine plaster and duloxetine, as monotherapy or in combination. Only publications in English were included. These criteria were applied to both citations retrieved from databases and hand-searching.

**Table 1:** Study inclusion and exclusion criteria

Criteria	Included	Excluded
Type of study	All study designs, both prospective and retrospective (except case studies for one patient)	Case studies for one patient

Population	Adult patients NeP due to any cause (central or peripheral) Treatment-refractory NeP (as defined in the included studies)	Studies in children Treatment naive patients

Study size	Any	None

Trial length	Any	None

Interventions	Pregabalin Lidocaine plaster Duloxetine	Other treatment for NeP

Comparator	Any/None	-

Language	English language only	Non-English language

### Study procedures

A rigorous systematic review process was conducted in accordance with the QUORUM guidelines [13]. A team of reviewers independently determined the eligibility of each publication by applying the inclusion/exclusion criteria. Each publication was reviewed by two reviewers, and any discrepancies in the decision for inclusion between these reviewers were resolved by a third reviewer. As several studies reported data in more than one publication, all publications of a single study were compiled into a single entry in the data extraction grid to avoid double counting of patient data. All efficacy, safety and tolerability data from eligible publications were extracted into a bespoke systematic review database. All data were double-extracted in parallel by two reviewers and any discrepancies were resolved by a third reviewer.

### Critical appraisal

A qualitative critical appraisal was carried out to assess the methodological quality and appropriateness of study design of each included study. A bespoke, qualitative critical appraisal tool was developed specifically for use in this review based on a number of published critical appraisal tools [14-17]. The development of a new critical appraisal tool was deemed necessary after assessing current instruments. The Jadad scale [18] which is often used in quality appraisal of randomised controlled trials (RCTs) was unsuitable for this review as the majority of included studies are not RCTs. A checklist for the assessment of the methodological quality both of randomised and non-randomised studies of health care interventions developed by Downs and Black was found to be more appropriate [19]. However, this tool consists of a detailed questionnaire, which was inappropriate for the brief content of several of the included studies only available as conference abstracts. Hence a new scale was developed which qualitatively covers the key topics covered in the Downs questionnaire (reporting, bias and confounding effects, usefulness to the study question-see Table 2).

**Table 2:** Critical appraisal

Reporting	Did the study address a clearly defined issue?
	Did the authors use an appropriate method to answer the review question?
	Are the main outcomes, patient population and interventions tested clearly described?
	Are losses to follow-up reported and clearly described?
Bias and confounding effects	Were the patients recruited in an acceptable way?
	Was any attempt to randomise or blind patents or investigators reported?
	Have the authors identified all/any of the confounding factors?
	Was the follow-up of patients complete and long enough?

Usefulness of the study	Are the results useful to answer the review question?
	Do the results fit with the evidence from other studies?

### Analysis

No meta-analysis or quantitative analysis was possible due to significant heterogeneity between included studies in terms of study design and size, and patient population (types of NeP included). Further, the inconsistencies in outcomes reported and the paucity of statistical data prevented quantitative meta-analysis. The review therefore consists of a qualitative assessment and narrative analysis to compare the studies.

## Results

### Trial Flow

**Figure 1:**
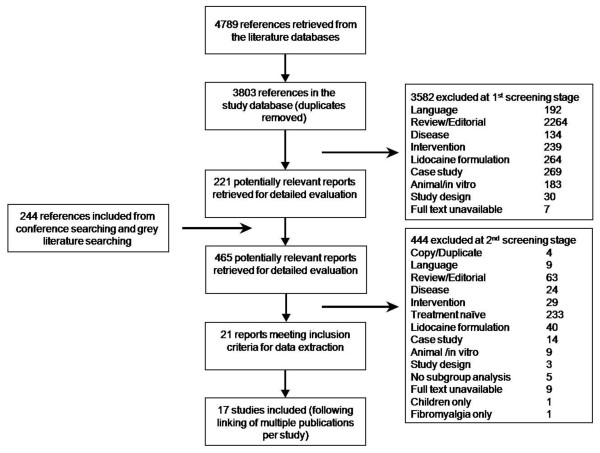
**Trial flow**. * Includes conference searching, Google scholar and Open SIGLE. Approximately 3500 references were reviewed in Open SIGLE and approximately 5500 references were reviewed in Google scholar.

Of the 4789 references retrieved from the literature databases, and 244 references retrieved from the hand searching, 21 publications met the inclusion criteria (Figure 1). Following linking of multiple publications per study, 17 studies were included. Reasons for study exclusion are presented in Figure 1.

### Trial Characteristics

The characteristics of the 17 studies included in the review are presented in Table 3. Seven of the studies were published solely as conference abstracts, while the remaining studies were available as full journal publications at the time of searching in December 2008.

**Table 3:** Summary of included trials

Study reference	Study location	Type of trial*	Study duration	No. pts	Treatment and dose	Study population	% pts refractory	Reporting and definition of refractory
Pregabalin								

*Freynhagen 2007 *[27]	*Germany*	*Single-arm trial*	*4 weeks*	*55*	*Pregabalin titrated from 75-600 mg/day*	Polyneuropathy, chronic radiculopathy***, encephalitis disseminate, PHN, neuropathic cancer pain, acute herpes zoster infection, CRPS (CRPS II), other	*100%*	*Diagnosis stated by the referring physician as "refractory NeP", "intractable NeP", "problematic NeP" or similar wording*. *Definition not reported*.

*Obermann 2008 *[33]	*Germany*	*Single-arm trial*	*52 weeks*	*53*	*Pregabalin titrated from 50-75 mg/d* *Mean (SD) dose 245 (77) mg/d at day 14*	*TN with and without concomitant facial pain*.	*94%*	*All but three of patients had received prior therapy, therefore may be considered refractory*.

*Sommer 2007 *[34]	*Germany*	*Single-arm trial*	*Unclear (mean duration of continuous pregabalin intake 217 days)*	*19*	*Pregabalin 75 mg bid was titrated*. *Mean effective dose (SD); 305 (185) mg*	*Restless Leg Syndrome with; Polyneuropathy, small fibre neuropathy, neurinomata*.	*89%*	*All but two patients had received prior therapy, therefore may be considered refractory*. *Reasons for discontinuation of therapy were lack or loss of efficacy, side effects and/or augmentation*.

Ambesh 2008 [24] (A)	Unclear	Unclear, active controlled	Follow-up was 2-18 months	86	Gabapentin 300 mg tid Gabapentin 150 mg and Pregabalin 75 mg bid Pregabalin 150 mg bid	NeP.	100%	Patients are "resistant to current analgesic treatment regimens or conventional pain therapies". Patients with "intractable NeP". Definition not reported.

Allen 2005 [26] (A)	UK	Single-arm trial	Up to 6 months	18	Mean dose of pregabalin was 600 mg/day	NeP.	100%	Patients with NeP "inadequately controlled by gabapentin". Definition of refractory not reported.

Douglas 2008 [41] (A) [linked to Douglas 2006 [31]]	UK	Audit	Data reported for 3 months	30	Pregabalin dosing "according to BNF recommended standard regime"	NeP.	100%	Patients who "failed to respond to or who had been unable to tolerate first and second-line neuropathic pain agents".

Hanu-Cernat 2005 [32] (A)	UK	Audit	Not stated	47	Dosing not stated	Variety of NeP conditions.	100%	Patients with an "unsatisfactory response to drugs". Patients who were previously treated with gabapentin which "failed to relieve the symptoms" or where the "dose could not be escalated due to side-effects".

Stacey 2008 [29] [linked to D'Urso De Cruz 2005; D'Urso De Cruz 2005; Siffert 2005 [42-44]]	United States	Single-arm trial	65 weeks	81	Pregabalin 150 mg/day titrated up to a max of 600 mg/d	DPN, PHN**	100%	Patients refractory to at least 6 months of usual care for NeP. Definition of refractory; discontinuation of a medication due to the inadequate effectiveness after 2 weeks of treatment at the minimum recommended doses, intolerable adverse events, or both.

Toth 2007 [30] (A)	Unclear	Single-arm trial	Unclear (Av. treatment duration 26 weeks)	33	Average dose 375 mg of pregabalin	NeP due to PN.	30% (only data on refractory patients-i.e. non-responders to gabapentin was extracted)	Responders and non-responders to gabapentin. Definition of non-responders not reported.

**Lidocaine plaster**								

*Galer 1999 *[22]	*United States*	*RCT, placebo controlled* *Enriched enrolment design*	*4 weeks (28 days max.)*	*32*	*Lidocaine 5% plaster (700 mg/plaster) applied as 3 plasters per day to the PHN region* *Placebo plaster, 3-5 plasters/day (10 × 14 cm)*	*PHN*	*Unclear*	*Compassionate use protocol enrolling patients who were participants of a previous study of the lidocaine plaster who had requested open-label use and those who were "refractory PHN patients"*. *Definition of refractory not reported*.

*Hines 2002 *[21]	*United States*	*Case series*	*Not stated*	*4*	*Lidocaine plaster 5% (Lidoderm)*	*Low back pain (2 patients with neuropathic component, 1 patient unclear if neuropathic component; lumbar degenerative disc disease, L4-L5 arthrodesis, and complex regional pain syndrome type 2 in 1 patient)*	*100%*	*All patients had previous treatment. The definition of refractory varied across patients from inadequate control of symptoms to intolerant of treatment*.

*Devers 2000 *[28]	*United States*	*Single-arm trial*	*Mean duration 6.2 weeks*	*16*	*Up to 3 plasters directly to the painful area* *Wear plasters up to a max 12 hours/day*	*NeP due to: Postthoractomy, Stump neuroma, Intercostal neuralgia, Abdominal neuroma* Radiculopathy***, Meralgia paresthetica, CRPS type 1, Diabetic polyneuropathy, Postmastectomy	*100%*	*All patients had been enrolled in prior drug trials, of which were unsuccessful; "either resulted in intolerable side effects of inadequate partial pain relief"*.

*Robert 2005 *[25]*(A)*	*Unclear*	*Case series*	*Not stated*	*3*	*Lidocaine plaster 5%*	*Central NeP syndromes due to: Spinal injury, IV infusion of infliximab*	*100%*	*All patient had been treated with previous therapy, however all "patients continued to experience excruciating pain"*.

*Argyra 2005 *[35]*(A)*	*Unclear*	*Single-arm trial*	*Unclear (length of treatment 2 months-4 years, mean 18 months)*	*36*	*Lidocaine 5% plaster* *Two plasters used every 24 hours*	*PHN, Posthoracotomy syndrome, Post mastectomy pain, DPN, CRPS, Peripheral ischaemia due to autoimmune disease*	*100%*	*Patients "resistant to therapy"*.

*Galer 2004 *[20]	*United States*	*Single-arm trial*	*6 weeks*	*71*	*Lidocaine plaster 5% daily, max. 4 plasters*	*Low-back pain (enrolled patients with non-radicular LBP, who reported moderate-to-severe pain on the neuropathic pain scale at study enrolment)*	*100%*	*Patients with the "moderate to severe pain ... at baseline despite prn or stable doses" of previous treatment*.

*Galer 2003 *[36]	*Unclear*	*Survey*	*Not reported (mean length of plaster use 7.6 years)*	*20*	*Lidocaine 5% plaster; the mean number of plasters applied to the PHN region was 2.3/d (range 1-5/day)*	*PHN*	*100%*	*The conclusion states that this study assessed "long term pharmacotherapeutic for a refractory neuropathic pain condition". Subjects were offered "compassionate use of the lidocaine plaster"*.

Duloxetine								

*Restivo 2008 *[23]	*Greece*	*Unclear, active controlled*	*12 weeks*	*18*	*Duloxetine 60 mg/day* *Duloxetine 120 mg/day*	*TN*	*100%*	*Patient were "refractory to medical treatment"*. *Definition not reported*

*All trials are single arm, unless otherwise stated. The type of trial was as reported by the authors. (A) indicates that only a conference abstract was available. NR; Not reported. All studies were prospective in design, with the exception of two [21,32]. Studies which do not use the licensed dose of the intervention and/or do not study the use of the intervention in a licensed indication are italicised (UK license, as reported by the European Medicines Agency (EMA) for pregabalin and duloxetine and the MHRA UK license for the lidocaine plaster, which was licensed by country rather than centrally by the EMA). **Fibromyalgia patients were also enrolled in this study, however data for these patients were reported separately and were not extracted for this review. ***Patients with radiculopathy are assumed to have radiculopathy with a neuropathic component as the studies state that patients with neuropathic conditions are included.

**Figure 2:**
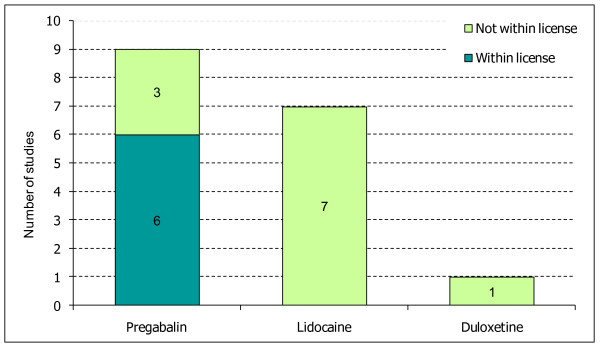
**Number of included trials within the UK license per treatment**.

Nine studies were identified in refractory NeP patients for pregabalin, seven for the lidocaine plaster and one study was available for duloxetine (Figure 2). Six studies, all assessing pregabalin were within the UK license, as reported by the European Medicines Agency (EMA) for pregabalin and duloxetine and the MHRA UK license for the lidocaine plaster, which was licensed by country rather than centrally by the EMA.

The types of NeP examined in the included studies were diverse (Table 3 and Table 4). Pregabalin was trialled in a variety of refractory conditions including DPN, postherpetic neuralgia (PHN), TN and CRPS. The lidocaine plaster was trialled additionally in lower back pain with a neuropathic component, but not in TN. Of the two lidocaine plaster trials including patients with lower back pain, one trial enrolled patients with non-radicular lower back pain (LBP), who reported moderate-to-severe pain on the neuropathic pain scale at study enrolment [20]. The other enrolled only four patients of which two patients had back pain with a neuropathic component; in one patient the presence of this component was unclear and the final patient had CRPS [21]. Duloxetine was only studied in TN.

**Table 4:** NeP sub-types studied in the included trials

Treatment	Licensed indication	Types of NeP studied within license	Types of NeP studied outside of license
Pregabalin	Peripheral and central NeP	• NeP (variety)	
		• Polyneuropathy	
		• Chronic radiculopathy with a neuropathic component	
		• PHN	
		• Diabetic peripheral NeP	
		• Neuropathic cancer pain	
		• CRPS (CRPS II)	
		• TN	
		• Restless Leg Syndrome with NeP	

Lidocaine plaster	PHN	• PHN	• Lower back pain with a neuropathic component
			• CRPS
			• NeP due to
			• Postthoractomy
			• Stump neuroma
			• Intercostal neuralgia
			• Abdominal neuroma
			• Radiculopathy
			• Meralgia paresthetica
			• Diabetic polyneuropathy
			• Postmastectomy
			• Peripheral ischemia

Duloxetine	DPN	-	• TN

Although a number of different types of studies were identified, including audits, case series and active controlled trials, the majority of included studies were single arm trials (Table 3). No head-to-head studies comparing the included treatments of pregabalin, duloxetine and the lidocaine plaster were found. Only one RCT (for the lidocaine plaster) was identified, which was crossover in design [22]. One active controlled trial compared two doses of duloxetine [23], while the other compared pregabalin to gabapentin [24]. Study duration varied across the included studies.

Few studies reported detailed definitions of 'refractory' NeP and in some cases, no definition was provided (Table 3). Previous failed medications were not always reported, nor were concomitant medications taken by trial participants. Where reported, commonly permitted concomitant medications included anticonvulsants and mood stabilisers such as carbazapine, tricyclic antidepressants (TCAs) and gabapentin (Table 5). Eight of the nine pregabalin studies trialled the intervention in patients who were refractory to gabapentin.

**Table 5:** Summary of previous, concomitant and add-on therapy

Study reference	Previous medication	Concomitant/add-on medication
Pregabalin		

*Freynhagen 2007 *[27]	*Anticonvulsants (except pregabalin), Antidepressants* *Cannabinoids, Gabapentin, Muscle relaxants, NSAID/COX-2 inhibitor, Opioids*	*Analgesics, Non-pharmacological treatment*

*Obermann 2008 *[33]	*Carbamazepine, Gabapentin, Oxcarbazepine*	*Carbamazepine, Lamotrigine*

*Sommer 2007 *[34]	*Cabergoline, Gabapentin, L-DOPA, Pergolide*, *Pramipexole, Roprinole, Roprinole + tilidine, Tilidine*	*Pramipexole + tilidine/naloxone* *Pramipexole + fentanyl patch*

Ambesh 2008 [24] (A)	Current analgesic treatment regimens, Conventional pain therapies	Amitriptyline

Allen 2005 [26] (A)	Gabapentin	NR

Douglas 2008 [41] (A) [linked to Douglas 2006 [31]]	Tricyclics, Gabapentin	NR

Hanu-Cernat 2005 [32] (A)	Gabapentin, Pregabalin	NR

Stacey 2008 [29] [linked to D'Urso De Cruz 2005; D'Urso De Cruz 2005; Siffert 2005 [42-44]]	Anticonvulsants, Gabapentin, NSAIDs/Cox IIs, Opoid analgesics, Pregabalin, SSRI, SNRI, Tramadol, Tricyclics	Tricyclics, Gabapentin

Toth 2007 [30] (A)	Gabapentin	NR

**Lidocaine plaster**		

*Galer 1999 *[22]	*Unclear; enriched enrolment design-patients either were treated with the lidocaine plaster and requested open-label use, or were refractory to previous treatment, although type of previous treatment was unclear*.	*Acetaminophen, Opiates, NSAIDs, Tricyclics*

*Hines 2002 *[21]	*Hydrocodone/acetaminophen, NSAIDs, Sertraline, Diazepam, Rofecoxib, Oxycodone, Progesterone therapy, Gabapentin, Atenolol, Ibuprofen, Opioid analgesics, Clonidine, Citalopram, Olanzapine, Lorazepam, Cyclobenzaprine, Hormone replacement therapy, Hydrocodone, Fluoxetine, Amitriptyline, Venlafaxine, Buspirone*	*Tramadol, Sustained-release diltiazem, Hormone replacement therapy, Triamterene, Amitriptyline, Sertraline, Diazepam, Rofecoxib* *Oxycodone controlled-release tablets, Tizanidine, Progesterone therapy, Verapamil, Clonidine, Baclofen* *Venlafaxine, Gabapentin*

*Devers 2000 *[28]	*Anticonvulsants, Eutectic mixture of local anaesthetics, Lidocaine, Mexiletine, Opoids, Prilocaine (EMLA) cream, Tricyclics*	*Amitriptyline, Acetamenophen, Carbamazepine, Fentanyl patch, Gabapentin, Hydromorphone, Lamotrigine, Methadone, Nortriptyline, Oxycodone, Paroxetine*.

*Robert 2005 *[25]*(A)*	*Combinations of conventional anti-neuropathic drugs:* *Anticonvulsants, Free radical scavengers, Ketamine, Opioids, Tricyclics*.	*NR*

*Argyra 2005 *[35]*(A)*	*NR*	*Lidocaine was tested as an add-on therapy, although other treatments were not reported*.

*Galer 2004 *[20]	*Cyclo-oxygenase-2 (COX-2) inhibitors, Non-selective NSAIDs, Gabapentin, Tramadol, Opioids*	*NR*

Galer 2003 [36]	Topical lidocaine plaster	Anticonvulsants, Acetaminophen, Conticosteroids, Opioids, Tricyclics.

Duloxetine		

*Restivo 2008 *[23]	*NR*	*NR*

### Trial Quality

Approximately half of the studies did not define or clearly report the study question. Withdrawals, an important aspect to determine complete follow-up of patients, were reported in the majority of pregabalin trials (7/9) but less frequently in the lidocaine plaster studies (4/7). The duloxetine study did not report withdrawals [23]. Bias and confounding factors were not addressed in many of the studies, most likely due to the brevity of information presented in the abstracts. Almost all of the included studies were single arm trials, which prevented direct and indirect comparisons of the interventions of interest.

### Comparison of efficacy outcomes

Mean pain scores were widely reported in the included trials, particularly for pregabalin (7 studies) (Table 6). In comparison, these outcomes were reported infrequently in trials of the lidocaine plaster and duloxetine (three and one studies respectively). All studies for pregabalin reported that the reduction in pain intensity was statistically significantly reduced following treatment; only one of the three studies reported statistical significance for this outcome in the lidocaine plaster studies. The duloxetine study reported that pain severity (measured by visual analogue scale (VAS)) was statistically significantly reduced at endpoint compared to baseline.

**Table 6:** Efficacy outcome reporting for each included intervention

	Intervention		
	**Pregabalin**	**Lidocaine plaster**	**Duloxetine**

Mean Pain Scores-No. studies (no. patients receiving the intervention)**^+ ^**reporting reduction in mean pain scores compared to baseline			

No. studies reporting data for any pain intensity outcome	7	3	1

McGill questionnaire	2* (109)		

Pain reduction measured by VRS	2* (81)		

Pain intensity measured by NRS	1* (30)	1NR (32)	

Pain intensity measured by BPI	2* (73)		

Pain score measured by NPS-10, NPS-8 and NPS-4		1* (71)	

Pain intensity measured by present pain intensity (PPI)	1* (81)		

Percentage pain score reduction		1NR (16)	

Pain severity measured by VAS			1* (18)

Quality of life-No. studies (no. patients receiving the intervention)**^+ ^**reporting improvements in quality of life compared to baseline			

No. studies reporting data for any quality of life outcome	4	1	0

SF-MPQ total, sensory and affective score	1*		

Quality of life measured by the SF-12	1* (55)		

Sleep interference measured by NRS	1* (55), 1NS (30)		

Quality of sleep measured by VRS	1* (28)		

Quality of sleep (instrument not reported)		1* (3)	

Inference of mood measured by VRS	1* (28)		

Daily activity measured by VRS	1* (28)		

Function interference measured by NRS	1* (30)		

Psychological stress measured by the Short Questionnaire on Current Burden	1* (55)		

Pain associated distress measured by NRS	1* (30)		

PGIC	1* (18)		

Responders-No. studies (no. patients receiving the intervention)**^+ ^**reporting data for outcome			

No. studies reporting data for any pain relief outcome	4	4	0

Complete pain relief	1 (53)	2 (48)	
	
range of percentages reported	25%	13-22%	

A lot of pain relief		2 (48)	
	
range of percentages reported		25-34%	

Moderate pain relief		2 (48)	
	
range of percentages reported		33-44%	

Pain reduction of ≥ 50%	3 (158)	2 (7)	
	
range of percentages reported	33-49%	100%**^a^**	

Pain reduction 10-50%	2 (105)	1 (33)	
	
range of percentages reported	17%**^b^**	NR**^c^**	

Non-responders	3 (132)	2 (52)	
	
range of percentages reported	26-46%	6-20%	

*Statistically significant; NS-not statistically significant; NR-statistical significance not reported.

^+^Numbers of patients receiving the intervention of interest.

^a^Two case studies of 3 and 4 patients: the former reported all patients achieved 50% reduction in pain score, the latter reported improvements in all patients in an inconsistent manner.

^b^One study reported that 4 of 24 patients achieved pain relief of 10-25%. The second study reported 38 out of 78 evaluable patients experienced ≥ 30% reduction in pain from baseline.

^c^One study stated that patients experienced pain relief of 10%-50%, however it was unclear whether this referred to all patients.

The proportion of responders was well reported for both pregabalin and the lidocaine plaster (Table 6); but there is considerable variation between trials, which may be explained by the variety of study designs identified. The duloxetine study did not report this outcome. The percentage of patients achieving complete pain relief was similar for both pregabalin and lidocaine plaster (25% and 13%-22% respectively). In comparison the percentage of patients achieving pain reduction of at least 50% varied greatly; most likely due to the diversity of trial designs. A similar trend was observed for the percentage of patients achieving a pain reduction of between 10% and 50%. The number of patients who did not respond to treatment ranged from 6% to 46%.

### Comparison of quality of life outcomes

**Figure 3:**
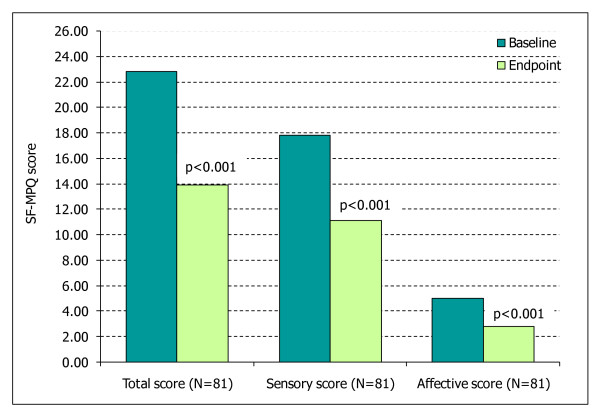
**SF-MPQ total, sensory and affective scores for pregabalin **[29] p values are compared to baseline

**Figure 4:**
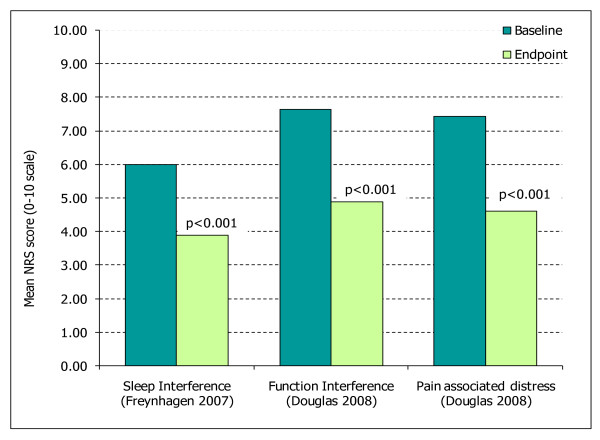
**Quality of life outcomes measured by the NRS (sleep and function interference, pain associated distress) for pregabalin **[22,41]. p values are compared to baseline. Freynhagen 2007, baseline N = 53, endpoint N = 50; Douglas 2008, baseline N = 30, endpoint N = 30

A substantial quality of life data-set was available for pregabalin; four of the nine included studies reported data for these outcomes (Table 6). Other than one study reporting a significant improvement in quality of sleep, no quality of life outcomes were reported in studies of the lidocaine plaster [25] and no results were available for duloxetine. In comparison significant improvements for pregabalin were observed in several aspects of quality of life including; overall quality of life (SF-MPQ total, sensory and affective scores (Figure 3) and SF-12), function interference, sleep interference, interference of mood, daily activities and pain associated distress (Figure 4). One study assessing pregabalin also reported that significant improvements in patients' global impression of change (PGIC) were demonstrated [26].

Treatment satisfaction was reported for one trial of pregabalin and two trials for the lidocaine plaster. A case series study of four patients using the lidocaine plaster described one patient as "satisfied with his pain control". A second study of 20 patients, reported that the mean satisfaction with pain relief from the lidocaine plaster was 3.8 on a scale ranging from-5 (extremely dissatisfied) to +5 (extremely satisfied). One pregabalin study reported that over 60% of patients were satisfied with treatment, while 65% of physicians were satisfied with treatment [27].

### Efficacy in patient subgroups

Pregabalin has been demonstrated to be efficacious across a broad range of NeP conditions. Devers et al. Reported 13 out of 16 patients with a wide range of NeP conditions achieved at least moderate pain relief with the lidocaine plaster [28]. One study reported a subgroup analysis for DPN and PHN patients and observed that pregabalin effectively reduced pain intensity in both subgroups with similar magnitude [29].

Pregabalin showed efficacy benefits in patients with NeP inadequately controlled by gabapentin as 8 of the 9 pregabalin studies included patients, who had previously failed on gabapentin (Table 5). A number of these studies reported a positive response to pregabalin in patients previously treated with adequate doses of gabapentin [26,27,29-32].

### Safety

Adverse events (AEs) were poorly reported with only eight studies reporting data for the proportion of patients who experienced any AE; three studies for pregabalin, four for the lidocaine plaster and one for duloxetine (Table 7). The proportion of patients who experienced AEs varied greatly between the trials for each intervention, in total ranging from zero to 42%.

**Table 7:** Any AE

Study Name	No. of patients	No. with any AE	Comments
Pregabalin			

Obermann 2008	53	22	

Sommer 2007	7	4	

Hanu-Cernat 2005	24		Intolerable side effects occurred in five patients.

Lidocaine plaster			

Hines 2002	4	0	

Devers 2000	16	1	

Galer 1999	32		No significant difference between lidocaine patch and placebo (p ≥0.492) for AEs that were reported by at least 5% of the subjects in either treatment group.

Galer 2003	20	5	

Duloxetine			

Restivo 2008	18	3	For both treatment groups of duloxetine (60 mg/day and 120 mg/day).

The most commonly reported AEs associated with pregabalin treatment were dizziness (range 16-66%) and somnolence (range 15-40%) [27,29,33,34]. Application site reactions/papules (4-28%) and local erythema (14-15%) were commonly reported with lidocaine plaster administration [20,22,28,35,36]. No safety data for duloxetine regarding specific AEs were reported.

### Tolerability

Withdrawals were reported for pregabalin (six studies) and the lidocaine plaster (four studies). No data was available for duloxetine. The proportion of patients withdrawing from studies varied considerably. Total withdrawals ranged from as little as 5% up to 30% for pregabalin. For the lidocaine plaster the range was narrower from 6% up to 11%. Withdrawals due to AEs ranged from 5.5% to 14.3% for pregabalin and 2.8% to 8.5% for the lidocaine plaster.

AEs were the main reason for withdrawals for both treatments. AEs leading to withdrawals were linked closely to commonly reported AEs for each treatment. For example, common AEs leading to pregabalin withdrawal included dizziness, drowsiness and weight gain, while AEs leading to withdrawal from lidocaine plaster use included rash and skin redness. Approximately 5% of patients withdrew due to lack of efficacy for both treatments.

### Study Limitations

The findings of this review are from a systematic and comprehensive search of the literature, aiming to collate all evidence on the treatments in question in refractory NeP in an unbiased and thorough manner. We do however note that there are limitations to this review. These include the finding that there are only a small number of included studies identified, all of which are heterogeneous in terms of patient population and study design. The studies are generally small, of poor quality, and enrol a large number of different refractory NeP conditions. This therefore limits meaningful comparisons between treatments in order to determine relative effectiveness.

## Discussion

Since a considerable number of patients suffer persistent or refractory NeP, one might expect a larger evidence base to be available [4]. The findings of this comprehensive systematic review indicate little clinical evidence is available for the refractory treatment setting. Only seventeen studies met the inclusion criteria of which seven were available as conference abstracts only. When considering the three included treatments in this review the evidence base for pregabalin in the refractory NeP population is stronger than for the lidocaine plaster or duloxetine. This is particularly apparent when considering only studies within UK licensing.

The studies included in this review were heterogenous in their design and reported outcomes. Patient populations enrolled were highly variable, ranging from a specific condition such as TN or chronic lower back pain with a neuropathic component to a "wide variety of NeP conditions". Pregabalin and the lidocaine plaster were trialled in a wide variety of NeP conditions. The duloxetine study enrolled a specific TN patient group, limiting generalisability to a clinical practice setting [23]. Both within and between study differences in NeP types of included patients are important factors, which should be considered when drawing conclusions from the results of the review. Study durations varied considerably with long-term data on key efficacy outcomes available for pregabalin only.

Despite the lack of consistency in instruments used, efficacy data is widely available for both pregabalin and the lidocaine plaster. In contrast, only a concluding statement (available as a conference abstract only) provided efficacy data for duloxetine. Relative differences between the interventions cannot be determined owing to the lack of head-to-head trials, or indeed any comparator controls to permit indirect comparisons. The significance of reductions in pain score were reported widely for pregabalin (significant in 7 out of 7 studies), while only one of the three studies for the lidocaine plaster using this outcome measure reported significant reductions. Wide variation between trials hinders comparison of the proportion of responders between treatments. Although treatment efficacy was reported in studies of a wide variety of NeP types, only one study carried out subgroup analysis, demonstrating efficacy of pregabalin in both DPN and PHN.

Quality of life data available for pregabalin indicated significant improvements. Of note, observed quality of life improvements in this patient population appears consistent with improvements in a treatment naive patient population [37].

Since completing this review, only one of the included abstracts has been published as full text in a peer-reviewed journal. The full report of the study by Toth [38] included data for a greater number of patients compared to the original abstract, for both the gabapentin responder (32 versus 23 patients respectively) and non-responder groups (29 versus 10 patients respectively) who were subsequently treated with pregabalin. A non-responder to gabapentin was defined as a patient who was unable to achieve at least a 30% pain relief on the VAS). Patients were assessed for both pain (using a VAS) and quality of life (using the European Quality of Life-5 Domains (EQ-5D) instrument and VAS), with only results from the pain relief presented in the original abstract. Both gabapentin responder and non-responder groups experienced similar pain relief as reported in the original abstract (20-25%) following substitution by pregabalin. There was also a statistically significant improvement in the VAS measured and utility scale quality of life in both responders and non-responders compared to baseline (statistical significance for pain relief was not reported in the original abstract). No serious adverse events were reported for either medication. However, gabapentin non-responders discontinued pregabalin in more than 30% of cases due to inefficacy or adverse events. Data from the full publication further supports the quality of life evidence base for pregabalin reported in this systematic review.

Additionally, two studies have been published since this review was conducted that would meet the inclusion criteria [39,40]. Both studies examined the efficacy of treatments in a refractory TN patient population. All patients had been previously treated with carbamazepine. The Rustagi et al. study was a randomised, cross-over trial comparing the efficacy of pregabalin with lamotrigine and was published as a conference abstract [40]. The authors concluded that although the efficacy of pregabalin was comparable to lamotrigine, pregabalin was found to have better tolerability, which would increase patient compliance when compared with lamotrigine. The study by Pérez et al. was a secondary analysis of the LIDO study, which aimed to analyse the effect of pregabalin on longitudinal health and non-health resource utilisation [39]. This was a non-interventional, 12-week, prospective, multicentre observational study in Spain that recruited patients with refractory NeP (including DPN, PHN and TN). This study reported a subgroup analysis on 65 patients identified with TN that were refractory to previous analgesic therapy. Patients received pregabalin as a monotherapy or as add-on therapy; all patients were naive to pregabalin treatment. The authors concluded that pregabalin was an effective short-term treatment, showing improvements in PROs such as pain reduction, anxiety, depression and sleep disturbances. However the limitation of this study was the lack of a relevant comparator.

### Recommendations for future research

This review has highlighted the need for future, high quality trials, particularly "gold-standard" RCTs in this refractory patient population. Head-to-head trials would enable further, direct (and indirect) comparisons between treatments, as previously proposed [12]. Currently, the main evidence for these treatments in refractory NeP stems from single arm trials of lesser quality.

In addition, a proposed consensus on the definition of refractory NeP is required, since studies have been shown to present a wide variety of meanings for the term. Where definitions of refractory NeP were provided in the included studies, these focused on either lack of adequate pain relief of previous treatments (2 studies) or a combination of intolerability/lack of pain relief to previous treatments (6 studies). No studies only referred to intolerability when defining refractory NeP. Further, only one study reported information regarding the dose and duration of previous treatment, which is included in the proposed definition of pharmacoresistant NeP [30]. Such broad definitions can result in hugely diverse disease severities both within and between studies, further limiting the potential to compare between treatments. We recommend further research both from the clinical community and from further real-world studies of this patient population towards a consensus definition of refractory NeP.

## Conclusions

Little evidence is available relevant to the treatment of refractory neuropathic pain despite the clinical need. There is a notable lack of high-quality comparative studies. Studies across all treatments included were diverse, generally small and of poor quality. The currently available evidence base found all three treatments to be efficacious in treating patients with refractory NeP. However, it is evident that there is a need for future, high quality trials, particularly "gold-standard" RCTs in this refractory patient population. Further, additional research may prove valuable in progressing towards a consensus definition of refractory NeP.

## Abbreviations

AE: Adverse event; BPI: Brief Pain Inventory; CRPS: Complex regional pain syndrome; DPN: Diabetic peripheral neuropathy; EQ-5 D: European Quality of Life-5 Domains; HIV: Human Immunodeficiency Virus; LBP: Lower Back Pain; MeSH: Medical Subject Headings; NeP: Neuropathic pain; NR: Not reported; NRS: Numerical rating scale; NS: Not statistically significant; NSAID: Non-steroidal anti-inflammatory drugs; PGIC: Patients' global impression of change; PHN: Postherpetic neuralgia; PN: Peripheral neuropathy; PPI: Present pain intensity; RCT: Randomised controlled trial; SF-MPQ: Short form of the McGill Pain Questionnaire; SIGLE: System for Information on Grey Literature; TCA: Tricyclic antidepressant; TN: Trigeminal neuralgia; VAS: Visual analogue scale; VRS: Visual rating scale.

## Competing interests

Melanie Plested and Sangeeta Budhia are current employees of Heron Evidence Development Ltd. Heron Evidence Development Ltd performed this research under contract with Pfizer. The manuscript was funded entirely by Pfizer. Zahava Gabriel is a current employee of Pfizer.

## Authors' contributions

These authors contributed equally to this work. All three authors contributed to the conception and design of the systematic review and were involved in drafting and revising the manuscript. MP, SB and a wider review team at Heron were involved in the acquisition, analysis and interpretation of the data. All authors read and approved the final manuscript.

## Pre-publication history

The pre-publication history for this paper can be accessed here:

http://www.biomedcentral.com/1471-2377/10/116/prepub

## Supplementary Material

Additonal file 1 **Systematic review search strategy**. Click here for file
