# Supplementary material for: Pregabalin, the lidocaine plaster and duloxetine in patients with refractory neuropathic pain: a systematic review
Source: BMC Neurol. 2010 Nov 19;10:116. doi: 10.1186/1471-2377-10-116 (PMC3003252; doi:10.1186/1471-2377-10-116)
Supplement: Additonal file 1 — Systematic review search strategy. [file 1471-2377-10-116-S1.DOC]

# Additional files

**Search strategy**

Medline® In-process & other non-indexed citations and Ovid Medline®: 1950 to present

| # | Search History |
| --- | --- |
| 1 | Neuralgia, Postherpetic/ |
| 2 | (Postherpetic neuralgia or PHN).mp |
| 3 | (Neuropathic pain or CNP or PNP).mp |
| 4 | exp Mononeuropathies/ |
| 5 | exp Polyneuropathies/ |
| 6 | exp Diabetic Neuropathies/ |
| 7 | (painful diabetic peripheral neuropathy or diabetic neuropath$).mp. |
| 8 | exp Brachial Plexus Neuropathies/ |
| 9 | exp Amyloid Neuropathies/ |
| 10 | ($neuropath$ or neuropath$).mp. |
| 11 | exp Fibromyalgia/ |
| 12 | (Fibromyalgia syndrome or fibromyalgia).mp. |
| 13 | exp Pain, Intractable/ |
| 14 | chronic pain.mp. |
| 15 | painful polyneuropath$.mp. |
| 16 | exp Trigeminal Neuralgia/ |
| 17 | (trigeminal neuralgia or neuralgia).mp. |
| 18 | Post?stroke pain? |
| 19 | exp Complex Regional Pain Syndromes/ |
| 20 | (complex regional pain syndrome? or CRPS or Reflex Sympathetic Dystrophy or RSD or Reflex Neurovascular Dystrophy or RND or Sudeck?s Atrophy or Algodystrophy or Algoneurodystrophy or causalgia).mp. |
| 21 | Exp Back pain/ |
| 22 | exp Radiculopathy/ |
| 23 | Back pain or radiculopathy.mp |
| 24 | Or/1-23 |
| 25 | Pregabalin.mp |
| 26 | Lyrica.mp |
| 27 | Versatis.mp |
| 28 | Lidoderm.mp |
| 29 | Lignocaine.mp |
| 30 | Lidocaine.mp |
| 31 | Exp Lidocaine/ |
| 32 | (Lidocaine plaster? Or Lidocaine patch$).mp |
| 33 | Duloxetine.mp |
| 34 | Cymbalta.mp |
| 35 | Or/25-34 |
| 36 | 24 AND 35 |
| 37 | limit 36 to yr="1998 - 2009" |

Embase (1980 to 2008 week 03)

| # | Search History |
| --- | --- |
| 1 | exp POSTHERPETIC NEURALGIA/ |
| 2 | (Postherpetic neuralgia or PHN).mp |
| 3 | exp Neuropathic Pain/ |
| 4 | (Neuropathic pain or CNP or PNP).mp |
| 5 | exp MONONEUROPATHY/ |
| 6 | exp Polyneuropathy/ |
| 7 | exp Diabetic Neuropathy/ |
| 8 | (painful diabetic peripheral neuropathy or diabetic neuropath$ or DPN or PDNP or PDPN).mp. |
| 9 | exp Brachial Plexus Neuropathy/ |
| 10 | exp Amyloid Neuropathies/ |
| 11 | ($neuropath$ or neuropath$).mp. |
| 12 | exp Fibromyalgia/ |
| 13 | (Fibromyalgia syndrome or fibromyalgia).mp. |
| 14 | exp Intractable Pain/ |
| 15 | exp Chronic Pain/ |
| 16 | chronic pain.mp. |
| 17 | painful polyneuropath$.mp. |
| 18 | exp Trigeminus Neuralgia/ |
| 19 | (trigeminal neuralgia or neuralgia).mp. |
| 20 | Post?stroke pain? |
| 21 | exp Complex Regional Pain Syndrome/ |
| 22 | (complex regional pain syndrome? or CRPS or Reflex Sympathetic Dystrophy or RSD or Reflex Neurovascular Dystrophy or RND or Sudeck?s Atrophy or Algodystrophy or Algoneurodystrophy or causalgia).mp. |
| 23 | Back pain.mp |
| 24 | exp Radiculopathy/ |
| 25 | radiculopathy.mp |
| 26 | Or/1-25 |
| 27 | Pregabalin.mp |
| 28 | Lyrica.mp |
| 29 | Versatis.mp |
| 30 | Lidoderm.mp |
| 31 | Lignocaine.mp |
| 32 | Lidocaine.mp |
| 33 | Exp Lidocaine/ |
| 34 | (Lidocaine plaster? or Lidocaine plaster$).mp |
| 35 | Duloxetine.mp |
| 36 | Cymbalta.mp |
| 37 | Exp Pregabalin/ |
| 38 | Exp Duloxetine/ |
| 39 | Or/27-38 |
| 40 | 26 and 39 |
| 41 | limit 41 to yr="1998 - 2009" |

Cochrane Central

| ID | Search |
| --- | --- |
| #1 | Pregabalin |
| #2 | Lyrica |
| #3 | Lidoderm |
| #4 | Lignocaine |
| #5 | Lidocaine |
| #6 | Lidocaine plaster* OR Lidocaine patch* |
| #7 | Duloxetine |
| #8 | Cymbalta |
| #9 | [MeSH descriptor Lidocaine explode all trees](http://www3.interscience.wiley.com/cochrane/searchHistory?mode=runquery&qnum=9) |
| #10 | [(#1 OR #2 OR #3 OR #4 OR #5 OR #6 OR #7 OR #8 OR #9)](http://www3.interscience.wiley.com/cochrane/searchHistory?mode=runquery&qnum=10) |
| #11 | [MeSH descriptor Neuralgia, Postherpetic explode all trees](http://www3.interscience.wiley.com/cochrane/searchHistory?mode=runquery&qnum=11) |
| #12 | [post*herpetic neuralgia](http://www3.interscience.wiley.com/cochrane/searchHistory?mode=runquery&qnum=12) or PHN |
| #13 | [neuropathic pain](http://www3.interscience.wiley.com/cochrane/searchHistory?mode=runquery&qnum=13) or CNP or PNP |
| #14 | [MeSH descriptor Polyneuropathies explode all trees](http://www3.interscience.wiley.com/cochrane/searchHistory?mode=runquery&qnum=14) |
| #15 | [MeSH descriptor Mononeuropathies explode all trees](http://www3.interscience.wiley.com/cochrane/searchHistory?mode=runquery&qnum=15) |
| #16 | [MeSH descriptor Diabetic Neuropathies explode all trees](http://www3.interscience.wiley.com/cochrane/searchHistory?mode=runquery&qnum=16) |
| #17 | painful diabetic peripheral neuropathy or diabetic neuropath* or DPN or PDNP or PDPN |
| #18 | [MeSH descriptor Brachial Plexus Neuropathies explode all trees](http://www3.interscience.wiley.com/cochrane/searchHistory?mode=runquery&qnum=18) |
| #19 | [MeSH descriptor Amyloid Neuropathies explode all trees](http://www3.interscience.wiley.com/cochrane/searchHistory?mode=runquery&qnum=19) |
| #20 | [*neuropath* OR neuropath*](http://www3.interscience.wiley.com/cochrane/searchHistory?mode=runquery&qnum=20) |
| #21 | [MeSH descriptor Fibromyalgia explode all trees](http://www3.interscience.wiley.com/cochrane/searchHistory?mode=runquery&qnum=21) |
| #22 | Fibromyalgia syndrome or fibromyalgia |
| #23 | [MeSH descriptor Pain, Intractable explode all trees](http://www3.interscience.wiley.com/cochrane/searchHistory?mode=runquery&qnum=23) |
| #24 | [painful polyneuropath*](http://www3.interscience.wiley.com/cochrane/searchHistory?mode=runquery&qnum=24) |
| #25 | trigeminal neuralgia or neuralgia |
| #26 | Post*stroke pain |
| #27 | MeSH descriptor Complex Regional Pain Syndromes explode all trees |
| #28 | MeSH descriptor Causalgia explode all trees |
| #29 | MeSH descriptor Reflex Sympathetic Dystrophy explode all trees |
| #30 | complex regional pain syndrome* or CRPS or Reflex Sympathetic Dystrophy or RSD or Reflex Neurovascular Dystrophy or RND or Sudeck*s Atrophy or Algodystrophy or Algoneurodystrophy or causalgia |
| #31 | MeSH descriptor Back pain explode all trees |
| #32 | MeSH descriptor Radiculopathy explode all trees |
| #33 | Back pain or radiculopathy |
| #34 | [(#11 OR #12 OR #13 OR #14 OR #15 OR #16 OR #17 OR #18 OR #19 OR #20 OR #21 OR #22 OR #23 OR #24 OR #25 or #26 or #27 or #28 or #29 or #30 or #31 or #32 or #33)](http://www3.interscience.wiley.com/cochrane/searchHistory?mode=runquery&qnum=26) |
| #35 | (#10 AND #34), from 1998 to 2008 |
